# Primary Ovarian Carcinomas and Abdominal Metastasis Contain 4,6-Disulfated Chondroitin Sulfate Rich Regions, Which Provide Adhesive Properties to Tumour Cells

**DOI:** 10.1371/journal.pone.0111806

**Published:** 2014-11-05

**Authors:** Myrtille J. E. Vallen, Samuel Schmidt, Arie Oosterhof, Johan Bulten, Leon F. A. G. Massuger, Toin H. van Kuppevelt

**Affiliations:** 1 Department of Biochemistry, Radboud Institute for Molecular Life Sciences, Radboud University Medical Center, Nijmegen, The Netherlands; 2 Department of Obstetrics and Gynaecology, Radboud University Medical Center, Nijmegen, The Netherlands; 3 Department of Cell Biology, Nijmegen Center for Molecular Life Sciences, Radboud University Medical Center, Nijmegen, The Netherlands; 4 Department of Pathology, Radboud University Medical Center, Nijmegen, The Netherlands; University of Patras, Greece

## Abstract

High mortality in ovarian cancer patients is primarily caused through rapid metastasis of the tumour, but the underlying mechanisms are poorly understood. Glycosaminoglycans, are abundantly present in tumours and chondroitin sulfate-E (CSE), a highly 4,6-sulfated glycosaminoglycan, has been indicated to play a role in carcinogenesis. In this study we investigated the presence of CSE in ovarian cancer metastasis and studied its role in tumour cell adhesiveness and migration. CSE was studied immunohistochemically in primary ovarian carcinomas and abdominal metastases using the single chain antibody GD3G7. The role of CSE was studied in 2D (scratch assays) and 3D (collagen matrices, spheroids) systems using SKOV3 cells applying 1: overexpression of CSE by stable transfection with DNA encoding GalNAc4S-6 sulfotransferase, 2: enzymatic removal of CS, and 3: addition of CSE. In ovarian cancer tissue, CSE expression was predominantly seen in the stromal compartment of both primary ovarian carcinomas and metastases, with a comparable degree of intensity and extent. Overexpression of CSE disaccharide units by tumour cells increased their adhesive properties which was especially seen in tumour spheroid formation. Increased expression of CSE reduced cell migration. Addition of free CSE had similar effects. The data presented here indicate that CSE is associated with metastatic lesions and that it provides tumours with adhesive properties. CSE rich motifs are put forward as a potential target for ovarian cancer therapy.

## Introduction

Ovarian cancer is the fifth leading cause of cancer death in women worldwide. Each year this disease accounts for approximately 225,000 new patients and 140,000 deaths [1]. Despite advances in cytoreductive surgery and modern chemotherapy, five-year survival rates are not improving. This high lethality is primarily due to the fact that patients are diagnosed with advanced stage disease (FIGO III–IV), when the tumour is already widely spread [2], [3]. Unlike other tumours, haematogenous dissemination of ovarian cancer cells is rare. Instead, ovarian carcinomas mainly disseminate via the transcoelomic route. Tumour cells and cell aggregates (spheroids) are shed from the primary tumour into the peritoneal space, where they preferably seed and attach to the peritoneum and omentum [4], [5].

In order for ovarian cancer cells to establish metastatic depositions, they need to aggregate and attach to the mesothelial lining. These initial steps in ovarian cancer progression are still poorly understood [6] and only little is known about the molecules involved in ovarian cancer cell adhesion [7]. There is increasing evidence that molecules in the extracellular matrix (ECM) play a crucial role in adhesiveness, and that the tumour stroma is a key player in this respect. It has been indicated that tumour cells may even bring their own soil, thereby providing a specific tumour niche and facilitating metastasis [8].

The ECM is a highly organized three-dimensional structure which maintains tissue integrity and is actively involved in many important physiological and pathological processes e.g. the binding of growth factors and adapting mechanical properties [9]. Tumour cells are known to interfere with the normal biosynthesis of the ECM and as a consequence modify its biochemical composition [10]. Proteoglycans and glycosaminoglycans are major constituents of the ECM and the cell membrane, mediating cell-cell and cell-matrix interactions. Proteoglycans are a complex group of glycoproteins, which contain a core protein with one or more covalently bound sulfated glycosaminoglycan chains. Based on the chemical backbone of glycosaminoglycans a number of classes can be discerned including dermatan sulfate (DS)/chondroitin sulfate (CS), heparan sulfate (HS)/heparin and keratan sulfate (KS). Hyaluronan (HA) is a special class of glycosaminoglycans not bound to a protein core. Normal ovary contains large amounts of DS [11], [12], however, during ovarian carcinogenesis the predominant glycosaminoglycan reverts to CS [13], a phenomenon seen in various types of cancer [14], [15]. Within the class of CS a large heterogeneity exists due to the presence of differently sulfated disaccharide units including CSA (4-sulfated), CSC (6-sulfated), CSD (2,6-disulfated) and CSE (4,6-disulfated). In cancerous tissue alterations in the sulfation level and type of the disaccharide units have been observed [16]–[21].

Using an antibody recognizing a motif that is rich in CSE units (GD3G7), we recently showed strong upregulation of CSE in primary ovarian carcinomas which correlated with poor prognostic parameters such as high grade, and advanced FIGO stage [19]. Interference with CSE, either by blockage using GD3G7 or by enzymatic degradation of CS chains, strongly inhibited metastatic growth in a mouse Lewis lung carcinoma model [22]. In addition, murine osteosarcoma cells containing high levels of CSE formed more metastatic liver tumour nodules compared to cells with lower levels, an effect that could be inhibited by preincubation of cells with the GD3G7 antibody or by preadministration with CS rich in CSE units [23].

The final step in the biosynthesis of the CSE subunit depends on the enzyme *N*-acetyl galactosamine 4-sulfate 6-*O*-sulfotransferase (GalNAc4S-6ST), which forms CSE by transferring an additional sulfate group to CSA [24]. An increase of GalNAc4S-6ST mRNA has been observed in patients with colorectal and astrocytic tumours [25], [26]. In astrocytic tumours elevated levels of GalNAc4S-6ST mRNA correlated with poor patient outcome. Down regulation of the expression of GalNAc4S-6ST in a Lewis lung carcinoma cell line resulted in reduced adhesiveness of cells to ECM molecules and suppression of metastatic lung carcinoma [27].

In this study, we first examined CSE expression in primary ovarian carcinomas and ovarian metastasis in the peritoneal cavity. We then addressed the mechanistic role of CSE, a.o. by establishing SKOV3 cell lines stably transfected with DNA encoding GalNAc4S-6ST and studying adhesiveness and cell migration using 2D and 3D model systems.

## Materials and Methods

### Human tumour tissue and cell lines

Paraffin embedded tumour material from 25 ovarian cancer patients (serous subtype) was obtained from the archives of the Department of Pathology at Radboud University Medical Centre. From all patients we collected representative tumour slides including primary carcinoma and abdominal metastases (omental and peritoneal tumour depositions). All slides were reviewed by an experienced gyneco-pathologist (JB) using H&E sections. The age at diagnosis, CA125 level at diagnosis, menopausal status, FIGO stage [28], tumour grade [29] and primary debulking result (defined as complete or incomplete) were recorded (Table 1).

**Table 1:** General patient characteristics.

	Panel (n = 25)
**Age** (median, range)	57 (23–84)
**CA125 at diagnosis** (median, range)	433.9 (53.9–8203.0)
**Menopausal status**	
Pre-	9
Post-	16
**FIGO**	
I	-
II	2
III	20
IV	2
Unknown	1
**Grade**	
I	-
II	2
III	23
**Primary cytoreductive surgery**	
Complete	9
Incomplete	14
Not reported	2

The human ovarian cancer SKOV3 cell line was generously provided by Dr. L.G. Poels (Radboud University Medical Center). Originally this SKOV3 cell line was obtained from the American Type Culture Collection (ATCC, Manassas, VA). Cells were cultured and maintained in Dulbecco's modified Eagle's medium (DMEM) supplemented with 10% fetal calf serum (FCS) (Life Technologies Paisley, UK) and penicillin/streptomycin (100 U/ml). Cells were incubated in a humidified atmosphere containing 5% CO_2_ at 37°C.

### Immunohistochemistry

Five µm paraffin sections were immunohistochemically analysed according to the ABC-method as described previously [19]. In brief, sections were de-paraffinised in xylene, hydrated with graded ethanol and blocked for endogenous peroxidase activity. After rinsing in phosphate-buffered saline (PBS) containing 0.1% Tween-20 (PBS-T), sections were incubated in 0.1 M citrate buffer (pH 5.8) and blocked with 2% bovine serum albumin (BSA, fraction V, Sigma-Aldrich) in PBS-T. Next, sections were incubated successively with primary anti-CS antibodies, secondary mouse anti-VSV antibody P5D4, and tertiary biotinylated horse-anti-mouse IgG (Vector Laboratories Inc.). After incubation with ABC-reagents (Vectastain ABC anti-mouse-IgG kit, Vector Laboratories Inc.), bound antibodies were detected using 3-amino-9-ethylcarbazole (AEC). Sections were counterstained with haematoxylin and mounted with Kaiser's glycerol gelatin (Merck, Darmstadt, Germany). Different single chain CS-antibodies were applied, including IO3H10 (overall staining for CS) [30], GD3A12 (staining for dermatan sulfate) [11], and GD3G7 (staining for 4,6-disulfated CS (CSE)) [31].

For immunofluorescence of SKOV3 cell lines, cells were plated on 10-wells glass slides (Nutacon, Portsmouth, NH) and grown to confluency. Cells were washed with PBS and fixed with 100% ice cold methanol. After blocking with PBS-T and 1% BSA, sections were incubated with primary antibody for 60 min. Bound antibodies were detected by secondary anti-VSV tag mouse antibody P5D4 and visualized by a tertiary antibody step including goat antimouse IgG Alexa Fluor 488 (Invitrogen, Paisley, Scotland), Finally, cryosections were fixed in 100% ethanol, air-dried and mounted in mowiol-488 (Calbiochem, La Jolla, CA).

### Generation of stably transfected SKOV-3 cell lines with DNA encoding for GalNAc4S-6ST

pFLAG-CMV2-hGalNAc4S-6ST, generously provided by Prof. Osami Habuchi, (Kariya, Japan), pBluescript SK II (Stratagene, La Jolla, USA) and pIRES-EGFP (Invitrogen, Paisley, Scotland) were used to construct the pIRES2-EGFP-GalNAc4S-6ST plasmid. An intermediate step was incorporated in the cloning procedure to generate compatible restriction sites (S. 1). Restriction enzymes *EcoRI*, *HindIII and Xhol* were purchased from New England Biolabs, Beverly, MA. Transfection of the SKOV3 was performed using Lipofectamine™^ 2000 ^(Invitrogen, San Diego, CA) according to the manufacturer's protocol. Geneticine G418 (Invitrogen, San Diego, CA) resistant cell clones were isolated and expanded for further characterization. For comparative purposes we selected two clones (C5 and C6) containing an empty vector (pIRES2-EGFP(-)) and three clones (F5, F7 and F9) with full vector (pIRES2-EGFP-GalNAc4S-6ST).

To establish the incorporation of the pIRES2-EGFP-GalNAc4S-6ST into genomic DNA, total genomic DNA was isolated from SKOV3 cells using a Qiagen AllPrep DNA/RNA micro kit (Qiagen GmbH, Hilden, Germany) according to manufacturer's specifications. The concentration of DNA was determined using a Nanodrop 2000c spectrophotometer (NanoDrop products, Wilmington, DE, USA). Primer sets covering exon-exon boundaries were designed using Primer 3 [32] based on the reference sequence obtained from the UCSC human genome browser (http://genome.ucsc.edu/), assembly February 2009 (GRCh37/hg19). Two primer sets for human GalNAc4S-6ST and 1 for the housekeeping gene human acidic ribosomal phosphoprotein P0 (RPLP0) were used (Biolegio (Nijmegen, The Netherlands). The primer sequences were as follow: hGalNAc4S-6ST, 5′-GACGCAGGACTTCATCCAC-‘3 (forward), 5′-AGCAACAGTTTCTCATTCTTCG-‘3 (forward), 5′GCGGATTTATTCGAACTTGC-‘3 (reverse) and 5′-ATCAAAGCCTCCTGCTTCTC-‘3 (reverse), and for RPLP0, 5′-CACCATTGAAATCCTGAGTGATGT-‘3 (forward) and 5′-TGACCAGCCCAAAGGAGAAG-′3 (reverse). Real-Time quantitative polymerase chain reaction (QPCR) was carried out with 5 ng DNA, using CFX Real Time PCR detection system and Bio-Rad CFX manager software version 1.6 for quantification with Sybr Green and melting curve analysis (Bio-Rad Laboratories, Inc., Hercules, CA). Amplifications were performed starting with a 10 min template denaturation step at 95°C, followed by 40 cycles of denaturation at 95°C for 15 s and combined primer annealing/extension at 60°C for 60 s.

### Reverse phase-high performance liquid chromatography (RP-HPLC) disaccharide analysis

Cells were plated in T175 flasks, grown to 80–90% confluency and detached using ethylenediaminetetraacetic acid (EDTA) and a police man scraper to harvest extracellular matrix components. The preparation was incubated with chondroitinase-ABC (Sigma Aldrich, St. Louis, USA) in digestion buffer (250 mM Tris, 20 mM MgAc_2_, pH 8.0) at a final concentration of 1 IU/ml (37°C, 24 h). Samples were vacuum dried and reconstituted with 0.1 M aminoacridone (AMAC) solution in glacial acetic acid/DMSO (3∶17, v/v). 1 M NaCNBH_3_ was added and the samples were incubated for 6 h at 45^O^C. Finally, 50% (v/v) dimethyl sulfoxide was added and the samples were stored at −80^O^C. The final disaccharide composition of CS was evaluated by quantitative analysis of the AMAC labeled digests using RP-HPLC. A gradient elution was performed using a binary solvent system as described by Ambrosius *et al* [33]. Equilibration of the Waters X-Bridge Shield column (100×4.6 mm ID) took place with 98% 60 mM ammonium acetate (NH_4_Ac), pH 5.6 and 2% acetonitril for 7 min. After injection of the samples the following program was used: 1 min at equilibration condition, a linear gradient step to 4% acetonitril in two min, a separation gradient step to 15% acetonitril in 23 min, a washing step in 1 min to 60% acetonitril followed by another 5 min at 60% and returning to equilibration conditions in 1 min. Flow rate was 1.5 ml/min and monitoring was performed with a fluorescence detector (λ_ex_ 442 nm, λ_em_ 520 nm).

### 2D scratch assay

For 2D-migration analysis a scratch assay was applied. Transfected and not-transfected SKOV3 cells were detached by EDTA, resuspended in DMEM medium containing 10% FCS and plated onto 12-well plates (5×10^4^ per well). Cells were grown to confluency and subsequently starved in a serum free DMEM medium for 24 hrs. After starvation, one artificial gap (scratch) was made using a sterile plastic 10 µl micropipette tip. Next, tissue culture medium was removed and cells were washed in sterile PBS to eliminate detached cells and debris. Experiments were performed by incubating the cells with normal medium or normal medium supplemented with CSE (Seikagaku, Tokyo, Japan) (50 and 100 µg/ml). Cell migration was monitored taking digital photographs at different time-points with a phase contrast microscope. All experiments were performed three or more times.

### Spheroid formation, the ‘hanging’- drop method

In accordance with the predominant metastatic pattern of ovarian carcinomas in the abdomen, we studied SKOV3 cell behavior not only as single cells but also as tumour cell aggregates (spheroids). Multicellular spheroids are thought to represent an important way for tumour dissemination in ovarian cancer. In addition, compact spheroid formation provides a protective shield for tumour cells. For that purpose, cells from subconfluent cultures were detached with 2 mM EDTA and multicellular spheroids were generated using the hanging-drop method [34]. In brief, cells were resuspended in DMEM growth medium supplemented with 10% methylcellulose to a final cell concentration of ±4×10^4^ cells/ml and incubated in hanging droplets (25 µl) overnight. After cell aggregation, spheroids were washed in phosphate buffered saline (PBS) and used for migration assays. To further study the adhesive properties of CS wildtype SKOV3 cells in hanging drops were digested with specific glycosaminoglycan degrading enzymes including chondroitinase ABC (Sigma Aldrich, St. Louis, USA), chondroitinase B and chondroitinase AC (IBEX Technologies, Montreal, Canada).

### Spheroid cell migration in 3-D collagen matrices

Spheroids (±1×10^3^ cells) prepared from different SKOV3 clones were incorporated into 3D collagen matrices as described before [35], [36]. In brief, SKOV3 spheroids were suspended in a solution containing 1.6 mg/ml bovine collagen type I (Inamed Biomaterials, Fremond, CA), 7% minimal essential medium and 3% bicarbonate in DMEM. The mix was gently transferred into a 6 well plate and allowed to polymerize for 20–30 minutes in an incubator at 37°C with 5% CO_2_. After polymerization the freshly prepared gels were covered with DMEM medium.

To study the influence of CSE on tumour cell migration, a protocol for CS decoration of the fibrillar type I collagen was developed. For this, an intermediate step was incorporated in which CSE (final concentration either 50 or 100 ug/ml) was added to the MEM bicarbonate suspension prior to collagen addition ((S2A). Inclusion of this step resulted in collagen fibrils associated with (FITC-labeled) CSE (AMS biotechnology, Abington UK), as demonstrated with confocal microscopy (S2B). To our knowledge, this is the first time a complex re-assembled meshwork of collagen fibrils homogeneously decorated with a negatively charged glycosaminoglycans is visualized. SKOV3 cell migration was microscopically recorded with an inverted microscope (Leitz Fluovert, Cologne, Germany) at 37°C and photographs were taken at time points 0, 12 and 24 hrs. All experiments were performed three or more times.

Cell viability was checked for single cells contained in DMEM supplemented with 50 or 100 µg/ml CSE using WST-1 analysis and cell counting based on nigrosin staining. Both CSE concentrations did not significantly affect cell viability (data not shown).

## Results

### Chondroitin sulfate expression in primary and secondary ovarian tumours

The clinical characteristics of the patient panel are summarized in table 1. Median age at the time of diagnosis was 57 (range 23–84) and the majority of women was postmenopausal. Our study panel consisted solely of patients suffering from high grade (II–III), serous ovarian carcinomas.

**Figure 1:**
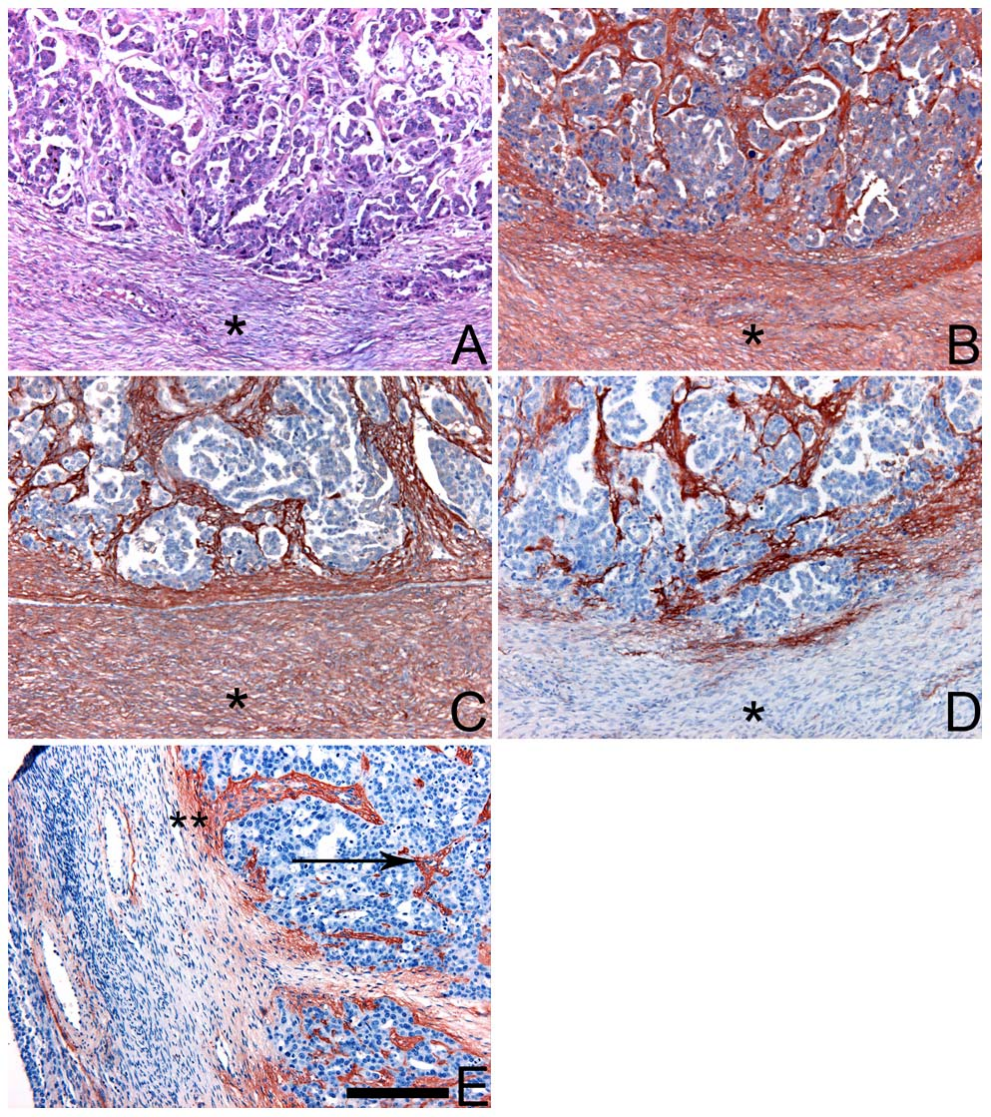
Strong staining for CSE in tumours. Sections of paraffin embedded primary serous ovarian carcinoma were stained for CS, DS and CSE using antibodies IO3H10, GD3A12 and GD3G7, respectively. A) Haematoxylin-Eosin staining showing tumourous versus non-tumourous regions. Non-tumourous regions are indicated with an asterix (_*_). B–E) Overall staining for CS (B: antibody IO3H10), DS (C:antibody GD3A12) and CSE (D, E antibody GD3G7). Note overall staining for CS and DS, but strong CSE staining in intratumoural stroma, including small tumour capillaries (E, arrows). Also note mild staining of large blood vessels in non-tumourous areas (E) Staining for CSE indicated a gradient of CSE at the boundary of tumour and non-tumourous tissue (_**_). Bar: 100 µm.

CS and DS are present throughout both the tumoural and non-tumoural stroma. This was demonstrated using antibodies IO3H10 (overall CS staining) and GD3A12 (overall DS staining) (fig. 1B and C) [11], [30]. We also applied antibody GD3G7 which defines a specific epitope harboring contiguous 4,6 disulfated (CSE) disaccharide units, a rare CS modification. In accordance with our previous studies [19], [31], GD3G7 expression in ovarian carcinomas was predominantly seen in the intratumoural stroma, the basement membrane zone underlying tumour cells, and the region surrounding (pathological) blood vessels. A CSE gradient was seen at places where tumour tissue is adjacent to non-tumourous tissue (fig. 1D and E).

**Figure 2:**
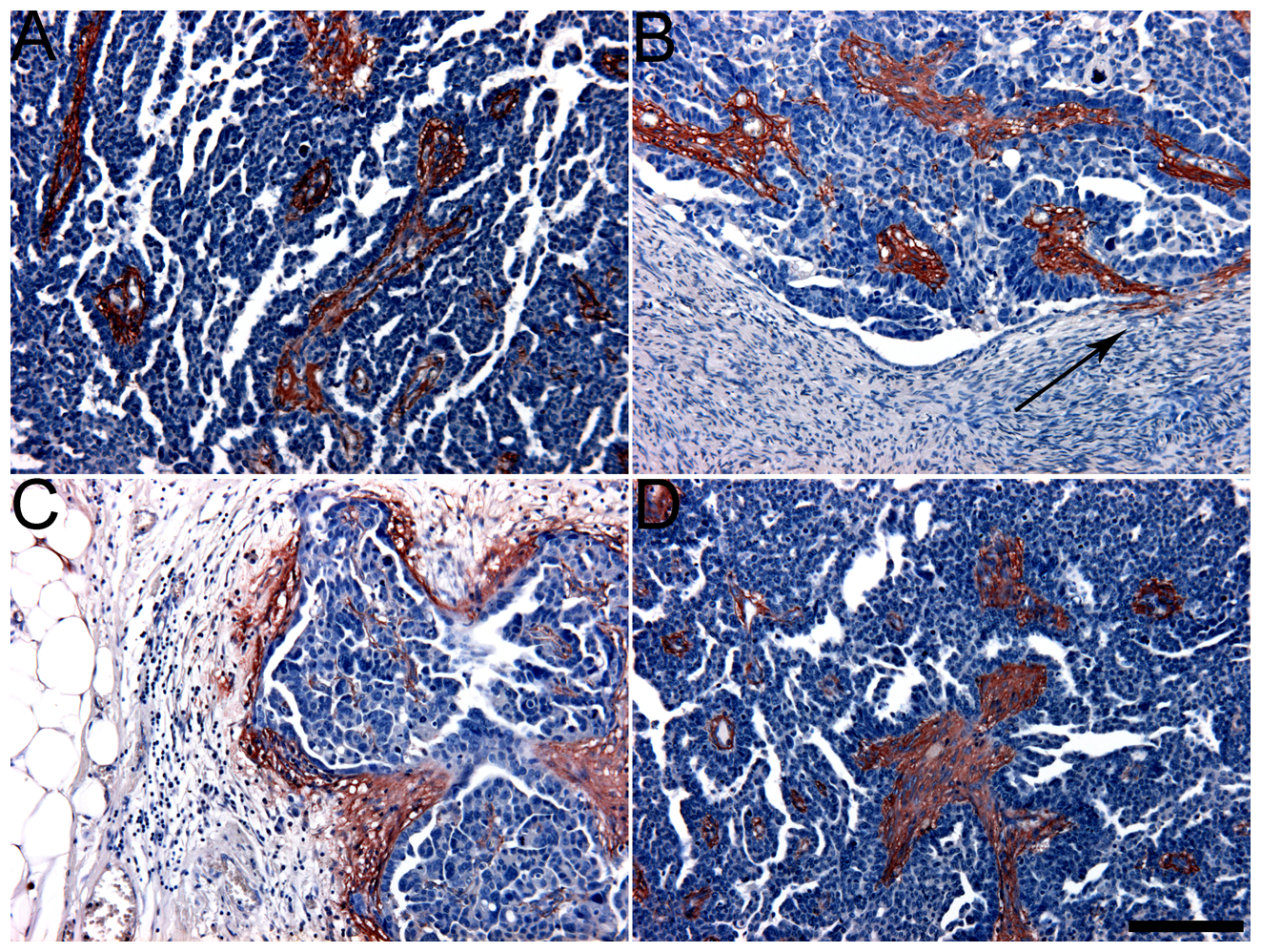
Strong staining for CSE in transcoelomic, ovarian metastasis (antibody GD3G7). A) Primary tumour, B) contralateral ovary, C) omentum, D) Douglas' pouch all displayed strong staining for CSE. Note the staining at sites where the mesothelial lining and its underlying basal membrane were associated with intruding tumour cells (B, arrow). Bar: 100 µm.

In general, primary ovarian tumours and abdominal secondary tumours showed a similar staining pattern. Both intensity and extent of CSE staining were comparable. In figure 2 GD3G7 epitope expression is shown for a primary tumour (fig. 2A), and three secondary metastatic tumour depositions, one on the contralateral ovary (fig. 2B), one in the omentum (fig. 2C) and one in the Douglas pouch' (fig. 2D). All metastatic localizations were strongly positive for the GD3G7 epitope. Again, GD3G7 expression was predominantly present in the intratumoural stroma, the stroma directly surrounding tumour depositions, and small (tumour) blood vessels. When the mesothelial lining and its underlying basement membrane were associated with tumour cells, an evident strong staining with GD3G7 was seen (arrow fig. 2B).

### Expression of chondroitin sulfate-E by SKOV3 cells transfected with DNA encoding GalNAc4S-6STGalNAc4S-6ST

To study the effect of CSE on tumour cell behavior we transfected cells with DNA encoding GalNAc4S-6ST, the enzyme involved in the biosynthesis of 4,6-disulfated disaccharides. We selected three stably transfected clones with the vector containing GalNAc4S-6ST and two clones containing an empty vector. These cell lines and wild-type SKOV3 cells were analysed for the presence of CSE using antibody GD3G7 and by disaccharide analysis of CS.

**Figure 3:**
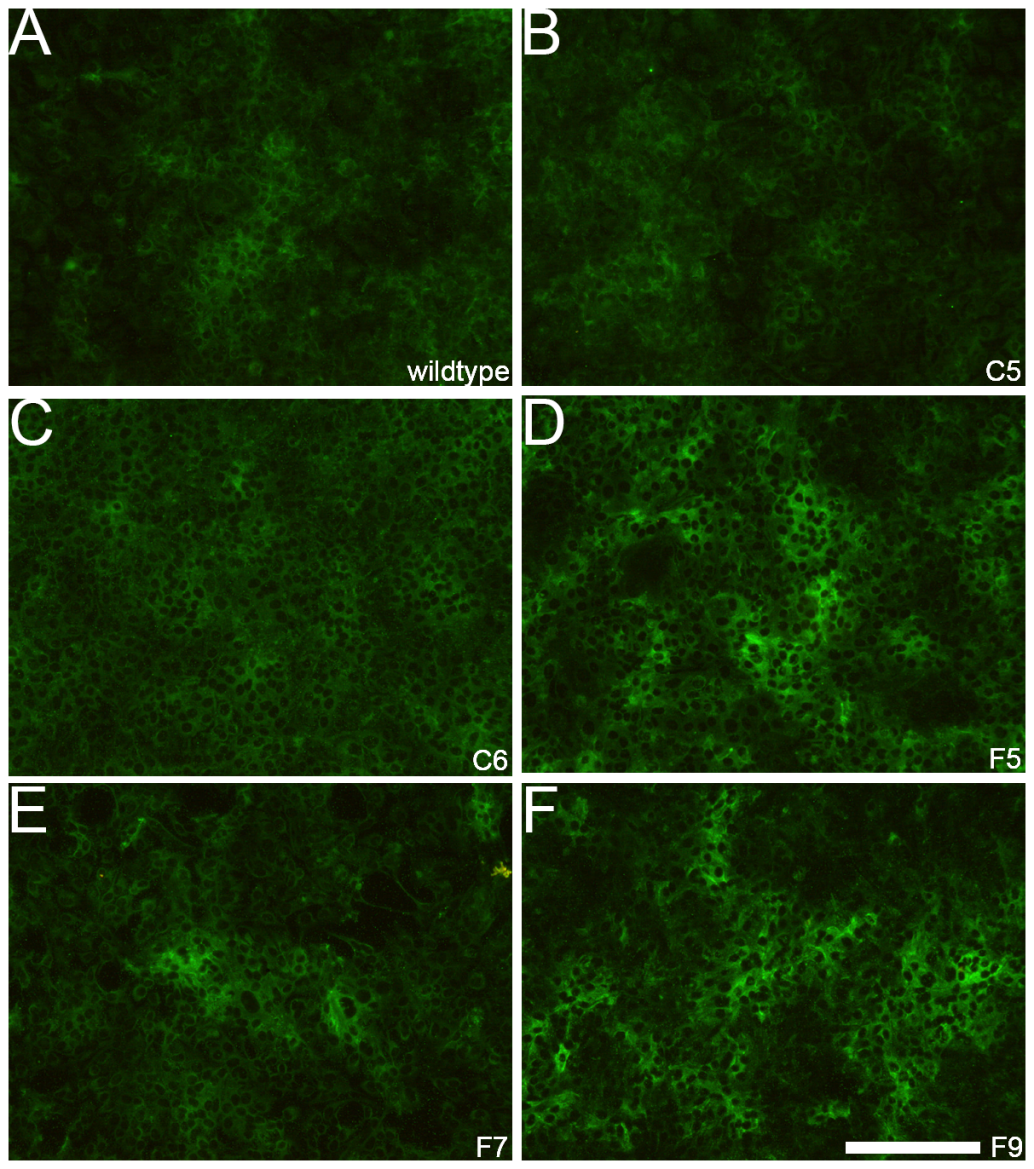
Increase of staining for CSE in SKOV3 cells transfected with GalNAc4S-6ST (antibody GD3G7). SKOV3 wildtype (A) and transfection control (empty vector) SKOV3(-) cells (C5 (B) and C6 (C)) showed weak (peri)cellular expression of CSE, whereas SKOV3 cells transfected with DNA encoding GalNAc4S-6ST (F5(D), F7(E), F9(F)) showed strong expression for CSE expression, especially in more dense regions. Bar: 100 µm.

Using confluent cells, the wildtype SKOV3 cells as well the cells containing the empty vector showed weak to moderate (peri)cellular staining with antibody GD3G7 (fig. 3A–C), whereas cells transfected with the DNA encoding GalNAc4S-6ST showed more abundant staining, including areas of very strong staining intensity (fig 3D–F).

**Figure 4:**
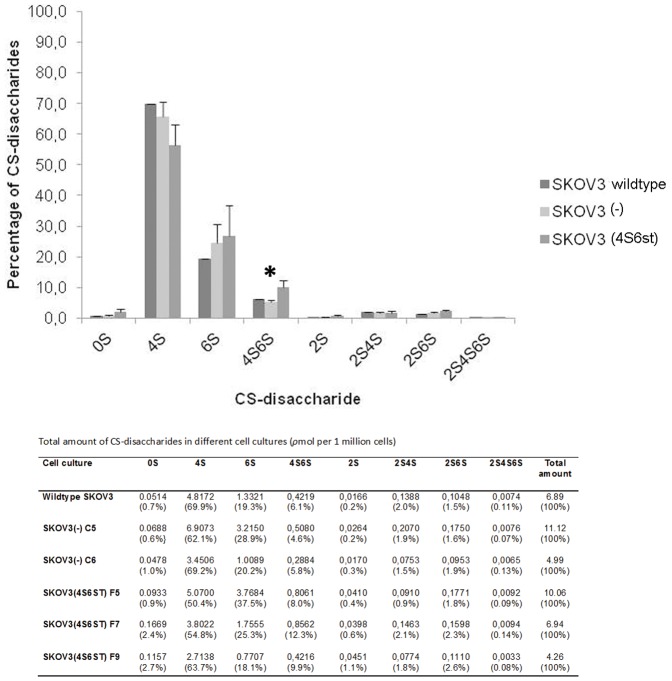
Reversed phase high performance liquid chromatography (RP-HPLC) for CS disaccharides. Chondroitin sulfate disaccharide composition of wild type, cells transfected with an empty vector (C5, C6), and cells transfected with a vector containing GalNAc4S-6ST (F5, F7, F9). Significantly higher CSE disaccharide content (*) was found in cells transfected with GalNAc4S-6ST (one-way ANOVA; level of significance p<0.05).

To analyse the disaccharide composition of CS we applied RP-HPLC with AMAC labelling. This technique allows separation and quantification of 8 different CS disaccharides based on their degree of sulfation. Overall, SKOV3 wildtype and empty vector clones were comparable for their CS disaccharide composition, including the CSE (4,6-di sulfated disaccharide) unit. When the CS disaccharide composition of full vector cell lines was compared with the control cell lines, CSE content was significantly elevated (p-value  = 0.026) (fig. 4), which was in line with the increased staining by GD3G7 (fig. 3). In addition, there was a tendency (p = 0.081), for a decrease in CSA disaccharide units (4S), which is the substrate of GalNAc4S-6ST.

### Chondroitin sulfate (E) provides adhesive properties

**Figure 5:**
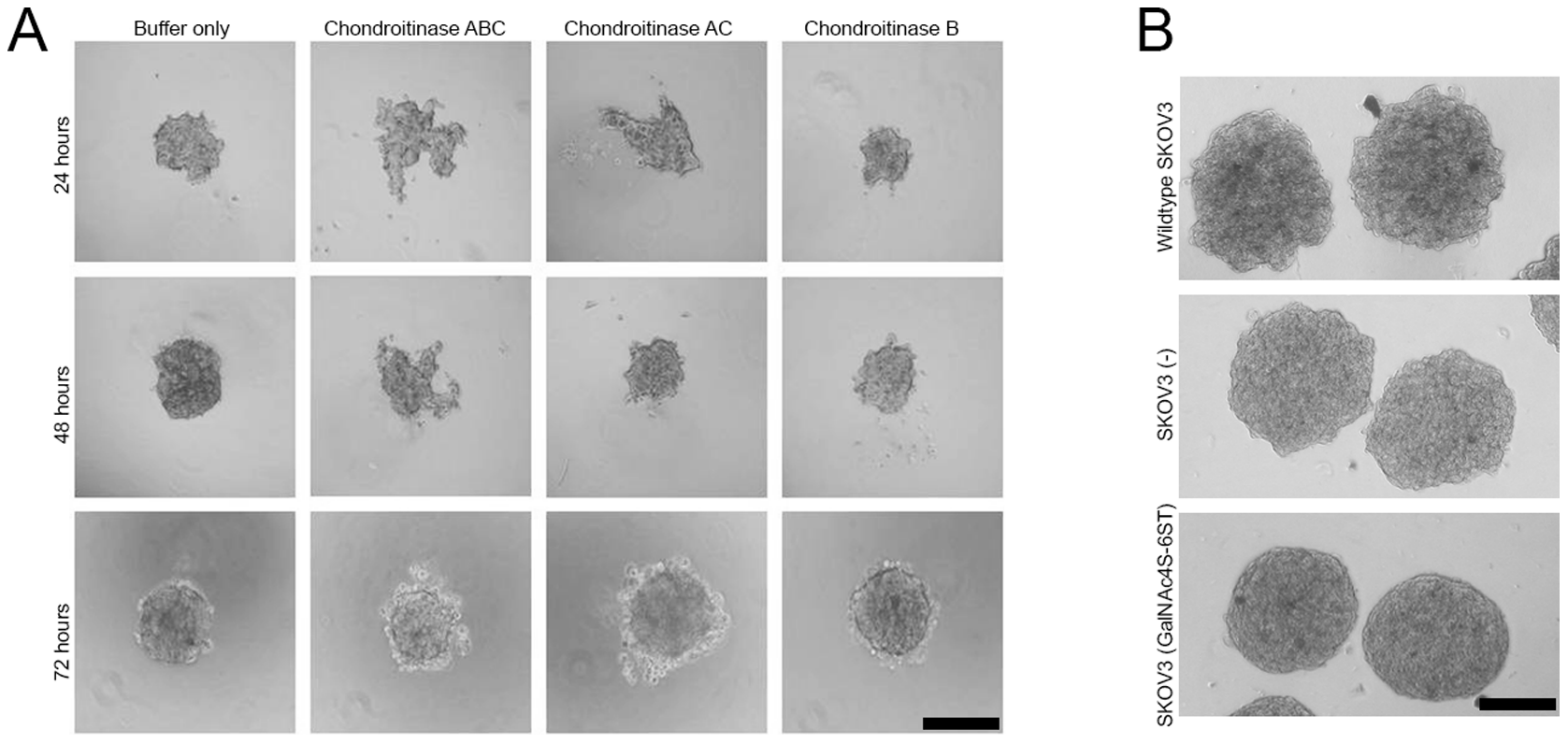
Spheroid formation by SKOV3 cells depends on chondroitin sulfate. A) Spheroid formation (tumour cell aggregation) was inhibited by enzymatic digestion using either of chondroitinase-ABC or –AC but not by chondroitinase B. Bar: 200 µm B) Spheroids formed by wild type SKOV3 cells and cells transfected with GalNAc4S-6ST and empty vector. Note the smooth and roundish appearance of GalNAc4S-6ST transfected cells. Bar: 100 µm.

Strong adhesive properties are an important survival tool of tumours. To investigate the role of CS in this respect, we prepared spheroids, cell aggregates often associated with tumour metastasis. Wildtype SKOV3 cells in hanging drops were digested with specific glycosaminoglycan degrading enzymes including chondroitinase ABC (cleaves both CS and DS), chondroitinase B (cleaves DS) and chondroitinase AC (cleaves CS). When either of chondroitinase ABC or AC was added, spheroid formation was inhibited for approximately 48 hours (fig. 5A). After 48 hours this effect disappeared, likely because of reduced enzyme activity. By contrast, chondroitinase B did not influence cell aggregation. Indicating that spheroid formation is CS but not DS dependent. To study the specific effect of CSE on spheroid formation, spheroids prepared from cells overexpressing CSE were evaluated. In comparison with to spheroids prepared from wild type cells or empty vector cells, CSE overexpressing spheroids displayed a more roundish appearance and showed a more smooth surface (fig. 5B), suggesting a more firm attraction of cells.

### Chondroitin sulfate E reduces cell migration

**Figure 6:**
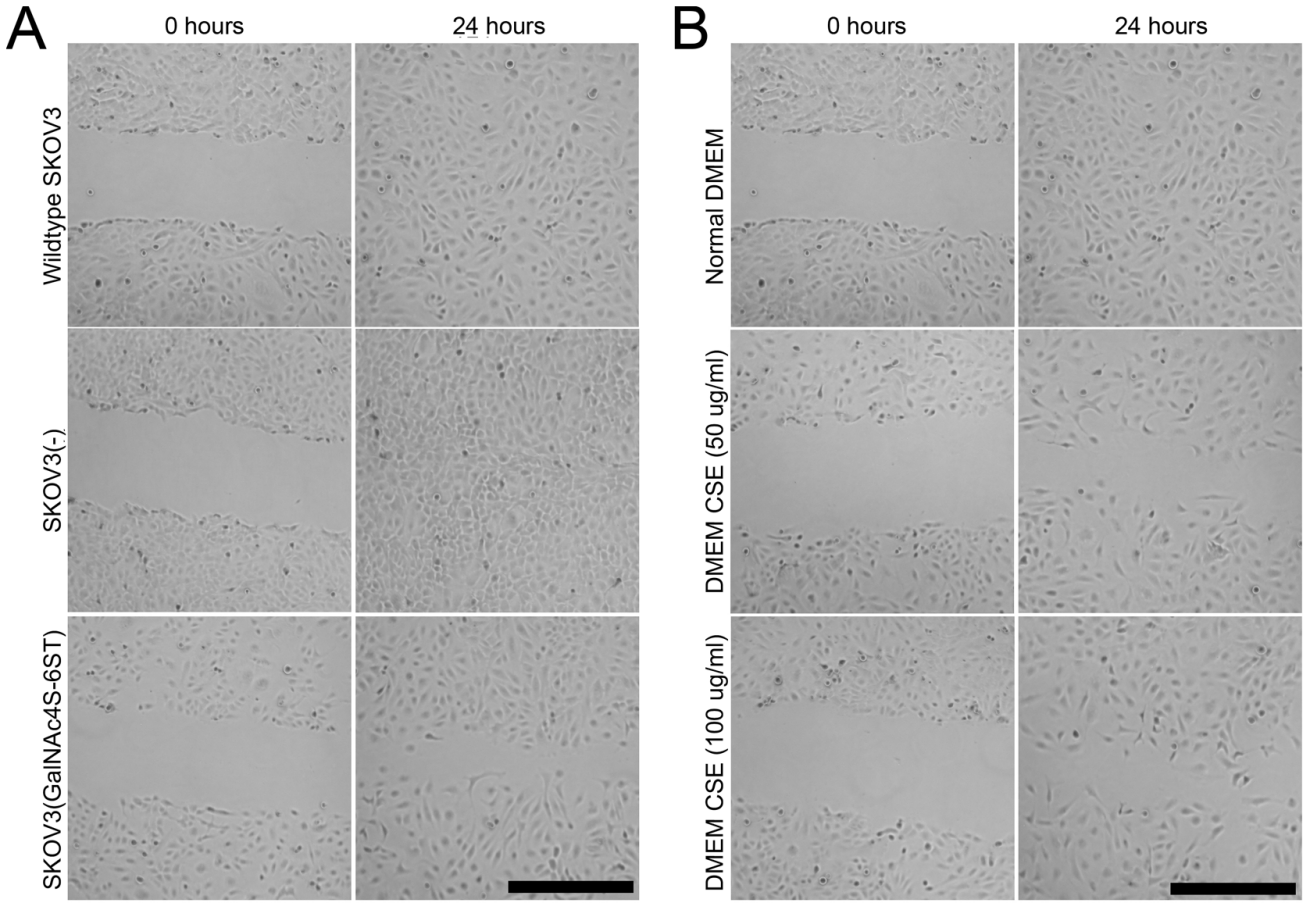
CSE inhibits artificial wound closure in 2D scratch assays. A) Wildtype SKOV3 cells and cell transfected with empty vector closed the artificial wound within 24 hours, whereas cells transfected with GalNAc4S-6ST cells did not. B) Addition of CSE to wildtype SKOV3 cells inhibited cell migration, showing limited gap-closure after 24 hours. Bar: 200 µm.

Since cell migration is an intermediate step in the metastatic process of ovarian cancer, the role of CSE was examined in 2D and 3D migration assays. We studied the migrative properties of wildtype and transfected SKOV3 cells in a 2D system using a scratch assay (fig. 6). Both wildtype SKOV3 cells and SKOV3 cells containing the empty vector closed the artificial wound within 24 hours. SKOV3 cells overexpressing CSE did not (fig. 6A). These cells closed the gap approximately 12 to 24 hours later. An inhibitory effect on migration was also seen when CSE was supplemented to the culture medium of wildtype SKOV3 cells (fig. 6B).

**Figure 7:**
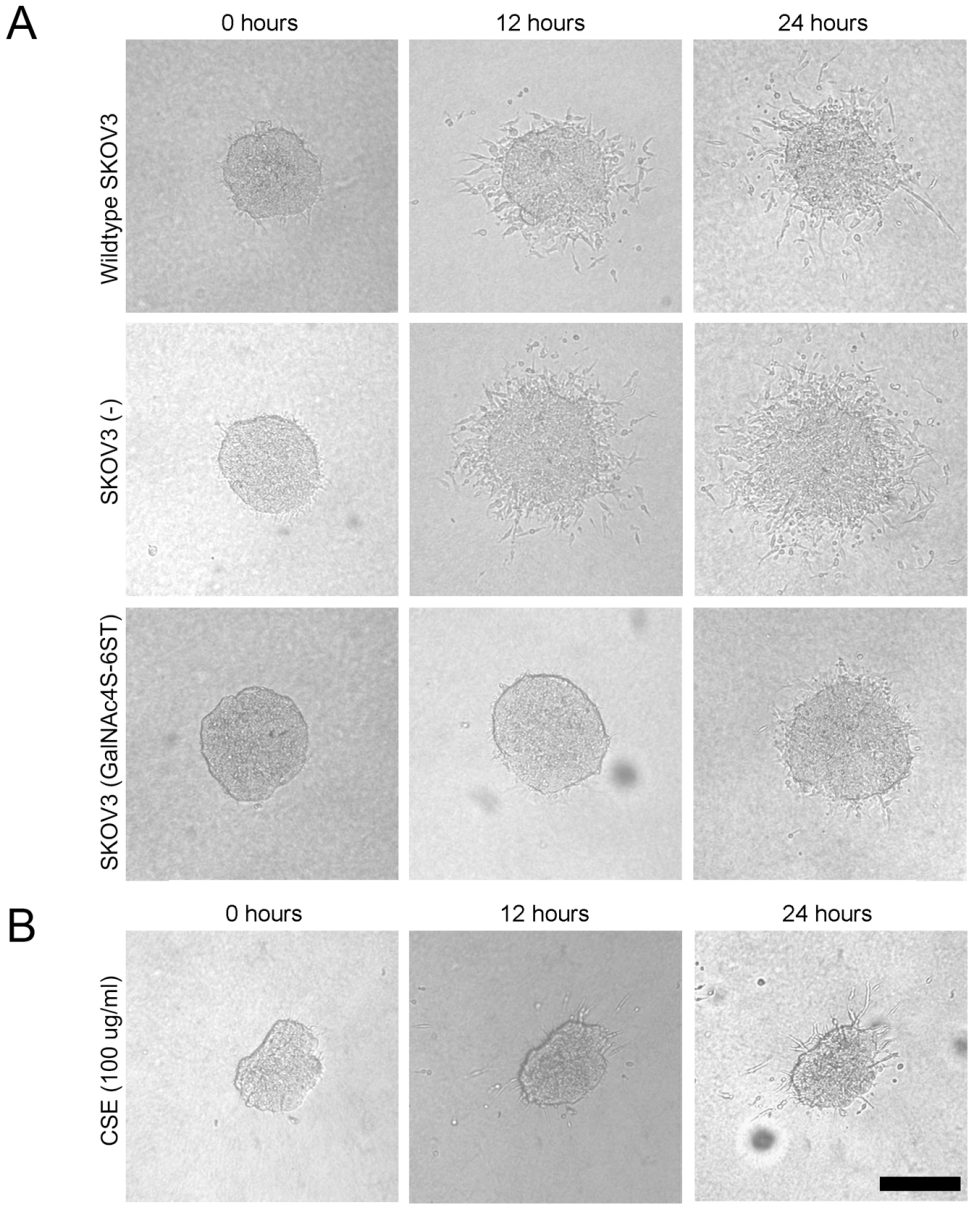
CSE inhibits SKOV3 cell migration out of spheroids in 3D collagen matrices. A) Wildtype and cells transfected with an empty vector (-) showed cell migration out of spheroids, whereas migration of cells transfected with GalNAc4S-6STwas almost absent. B) Strong restriction of migration of wildtype SKOV3 cells out of spheroids in CSE decorated collagen matrices. Bar: 200 µm.

To study the effect of CSE on cell migration in 3D systems, spheroids were prepared from wildtype and transfected SKOV3 cells and embedded in a collagenous matrix. Wildtype SKOV3 cells migrated out of the spheroid primarily as single cells and sometimes following collective strand formation. The same was seen for spheroids prepared from SKOV3 cells harboring an empty vector. However, SKOV3 cells overexpressing CSE were hardly able to migrate out of the spheroid, nor as single cells, nor as collective strands (fig. 7A). In an alternative approach, we prepared collagenous matrices in the presence of CSE, resulting in collagen fibrils associated with CSE. In such matrices wildtype SKOV3 cells showed very restricted cell migration (fig. 7B), further indicating the cell migrative inhibiting effect of CSE.

## Discussion

High lethality in ovarian cancer is primarily due to rapid metastasis of the tumour. In this study we observed manifest presence of CSE, a CS harbouring 4,6-disulfated disaccharides, in both primary and metastatic ovarian tumours, and we propose a functional role for CSE in tumour cell adhesion in ovarian cancer spheroids.

CS likely possesses a number of roles in the micro-environment of (tumour) cells and upregulation of highly sulfated CS (such as CSE) has been described for various tumours. Through the binding of glycosaminoglycans with effector molecules such as cytokines, chemokines, and growth factors [37], chondroitin sulfates (especially CSE) are able to regulate many cellular mechanisms in both physiological and pathological conditions, including proliferation and angiogenesis. The metastatic cascade involves detachment, adhesion and migration/invasion, and encompasses various interactions between tumour cells and components of the ECM [38].

Ovarian cancer dissemination predominantly occurs via intra-abdominal spread of the tumour. Exfoliated tumour cells are carried via peritoneal fluid or ascites to secondary sites in the abdominal cavity [39]. Spheroid formation may represent an important intermediate survival mechanism facilitating ovarian cancer dissemination. Compact formation of spheroids by ovarian cancer cells has been associated with more aggressive and invasive characteristics [40], [41]. In addition, decreased penetrance of immune cells and therapeutics provides spheroids with an important advantage for survival [42]. In this study, we found that CS has a role in spheroid formation, and overexpression of CSE disaccharide units improves the adhesive properties of tumour cells. Our results are in line with the finding that down-regulating of GalNAc4S-6ST in Lewis lung carcinoma cells reduced adhesion and restricted the formation of novel tumor noduli [27]. Like lung cancer, the majority of malignant tumours spread through hematogenous or lymphogenous pathways, and upregulation of CSE may play a role. Blood born metastatic tumour cells with adhesive properties are more likely to develop tumour noduli at secondary sites. In addition, the formation of a physical barrier (pericellular matrix containing CSE) may provide a protective environment against the immune system and chemotherapy.

*In vivo*, CSE expression is predominantly seen in the stromal compartment of both primary ovarian carcinomas and metastasis. The stromal compartment is actively involved in tumour progression and metastasis. The presence of large intratumoural stroma areas has been correlated with poor prognosis in ovarian cancer patients [43]. Tumour cells create a tumour-facilitating micro-environment by altering the pre-existent stroma. In addition, tumour cells create ECM during spheroid formation [44]. CS, as part of the tumour micro-environment, accumulates in the stromal compartment of many solid tumours, including ovarian carcinoma [13], [45]. The “seed and soil” hypothesis proposes that an appropriate host micro-environment (the soil) is needed for the optimal growth and spread of tumour cells (the seed) [46]. Recently, it was suggested that tumour cells might bring their own soil [8]. For this matter, spheroids may provide the ideal vehicle to bring soil (metastatic niche) to a secondary tumour site. This metastatic niche includes ECM components, growth factors and activated fibroblasts (CAFs), all active facilitators for tumour growth and metastasis formation [8]. Identifying adhesion molecules that are responsible for adherence (such as CSE) between cancer cells, stroma and CAFs may provide new targets for anti-metastasis therapy.

Strong CSE expression was observed at the “invasion front” of metastatic lesions (fig. 2). The upregulation of CSE at the secondary site may be a tumour cell induced modification of host cell ECM in such a way that it presents the appropriate soil for tumour growth and angiogenesis. The binding properties of CSE for a large variety of tumour associated growth factors such as FGF2 and VEGF, but also important adhesion molecules including N-cadherin and E-cadherin may be instrumental in this regard. [47], [48] In addition, CSE may be one of the adhesion molecules involved in the adherence between cancer cells and stroma. It was shown that pretreatment of ovarian cancer cells with the chondroitin sulfate cleaving enzyme chondroitinase ABC decreased cell adhesion to the ECM proteins fibronectin, type IV collagen and laminin, and mesothelial cells [49], [50].

Although tumour cells are the prime targets of cytostatic therapy, the tumour stroma has been suggested as an attractive target for cancer treatment as well. CSE, which is accumulated in tumour stroma, could be a specific target in this respect. Blockage, inhibition or enzymatic degradation of CSE could interfere with tumour cell aggregation, growth factor binding, and metastasis. Enzymatic degradation of CS by chondroitinase AC has been shown to inhibit melanoma proliferation and invasion [51]. However, conflicting data are available to the effect of enzymatic degradation of all chondroitin and dermatan sulfate by intra-tumoral injections of chondroitinase ABC. For instance, enzymatic degradation of *all* CS/DS by intra-tumoural injections of chondroitinase ABC results in the development of secondary tumours and increased lung metastases [52]; in solid Ehrlich ascites tumors, however, growth was restricted after chondroitinase ABC injection [53]. This suggests that targeting specific chondroitin sulfates, rather than all of them, is key to a potential anti-tumour strategy.

The data presented here indicate that a specific 4,6-disulfated CS motif (CSE) is associated with metastatic lesions and that this structural motif provides tumours with adhesive properties. If specifically addressed, the CS motif may be a potential target for ovarian cancer therapy.

## Supporting Information

Information S1 **Cloning strategy to obtain GalNAc4S-6ST containing pIRES2-EGFP plasmid.** A) pFLAG-CMV2-hGalNAc4S-6ST plasmid was double digested with restriction enzymes *EcoRI* and *HindIII* to obtain the GalNAc4S-6ST insert. The pBluescript SK II (-) vector was double digested with restriction enzymes *EcoRI* and *HindIII*, and the GalNAc4S-6ST insert was ligated vector, generating an intermediate vector. B) The intermediate vector and the pIRES2-EGFP plasmid were double digested with restriction enzymes *EcoRI* and *IXhoI*, *and* GalNAc4S-STt ligated into the pIRES2-EGFP plasmid.
(TIF)

Information S2 **Generation of 3D collagen matrices with homogeneously coated chondroitin sulfate E chains, and culturing of SKOV3 spheroids for cell migration studies.** A) The spheroids were embedded into a matrix of type I collagen as described previously [54], but with an additional step for administration of the chondroitin sulfate E (CSE, 1 mg/ml) into the minimum essential medium (MEM)/bicarbonate (BIC) solution before adding the bovine type I collagen. SKOV3 spheroids in DMEM medium were transfered into the collagen I/chondroitin sulfate E solution reaching a final concentration of collagen-spheroid suspension of 1.67 mg/mI. The suspension was quickly pre-polymerized for 5 minutes at 37°C, 5% CO_2_ and eventually allowed to polymerize at 37°C for 20–30 min (5% C0_2_) in a self-constructed cell migration chamber [54]. The type I collagen–chondroitin sulfate matrices were analyzed by using an Olympus FV1000 confocal laser scanning microscope excitation at 488 nm and emission detection of 520/50 nm (for FITC-labeled chondroitin sulfate) and confocal reflection contrast was used for detection of collagen fibers. For that, laser light (633 nm) at a low intensity was introduced into the sample. B) Confocal microscopy showing matrix decoration with chondroitin sulfate E (CSE). Upper row; non-decorated type I bovine collagen matrix. Left: Collagen reflection (white), middle: Background (green (FITC) channel), right: Overlay of reflection and background signal. Lower row; CSE-decorated bovine collagen I matrix. Left: Collagen reflection, middle: CSE-FITC (green (FITC) channel), right: Overlay of reflection and CSE signal.
(TIF)
