# Supplementary figures and images for: Primary Ovarian Carcinomas and Abdominal Metastasis Contain 4,6-Disulfated Chondroitin Sulfate Rich Regions, Which Provide Adhesive Properties to Tumour Cells
Source: PLoS One. 2014 Nov 5;9(11):e111806. doi: 10.1371/journal.pone.0111806 (PMC4221137; doi:10.1371/journal.pone.0111806)

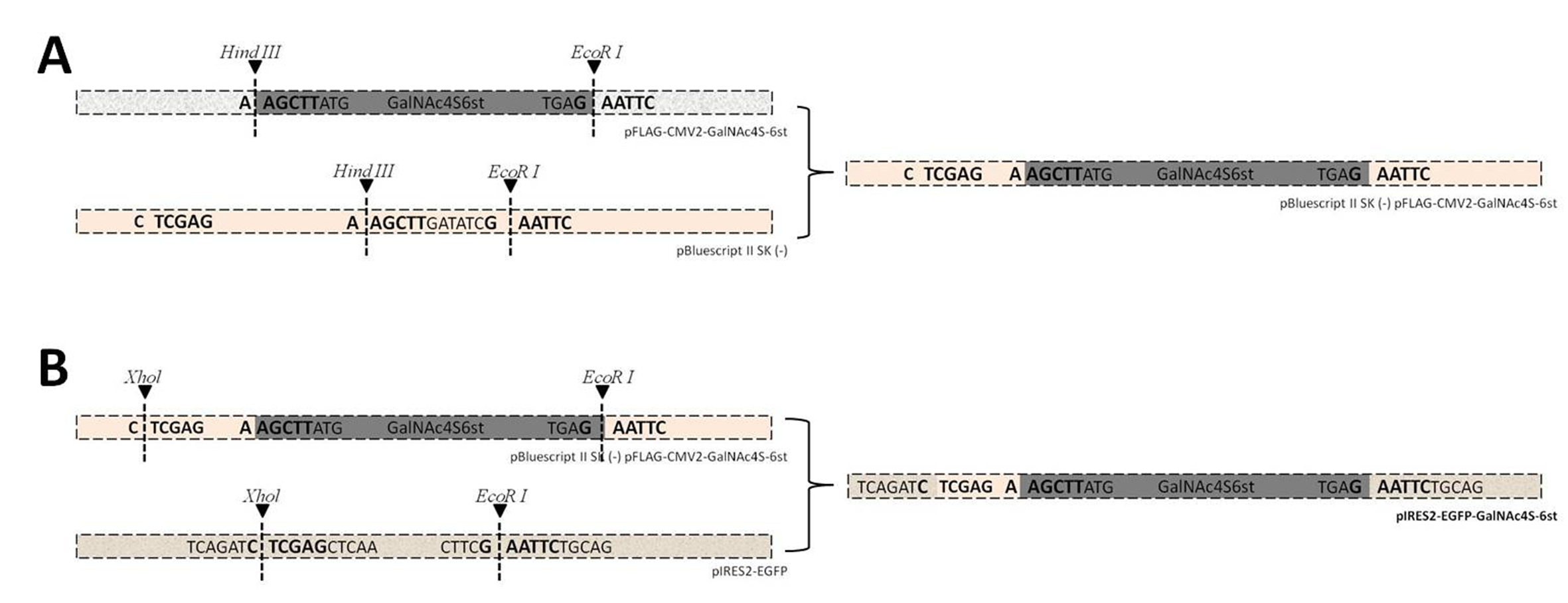

Supplement: Information S1 — Cloning strategy to obtain GalNAc4S-6ST containing pIRES2-EGFP plasmid. A) pFLAG-CMV2-hGalNAc4S-6ST plasmid was double digested with restriction enzymes EcoRI and HindIII to obtain the GalNAc4S-6ST insert. The pBluescript SK II (-) vector was double digested with restriction enzymes EcoRI and HindIII, and the GalNAc4S-6ST insert was ligated vector, generating an intermediate vector. B) The intermediate vector and the pIRES2-EGFP plasmid were double digested with restriction enzymes EcoRI and IXhoI, and GalNAc4S-STt ligated into the pIRES2-EGFP plasmid. (TIF) [file pone.0111806.s001.tif]

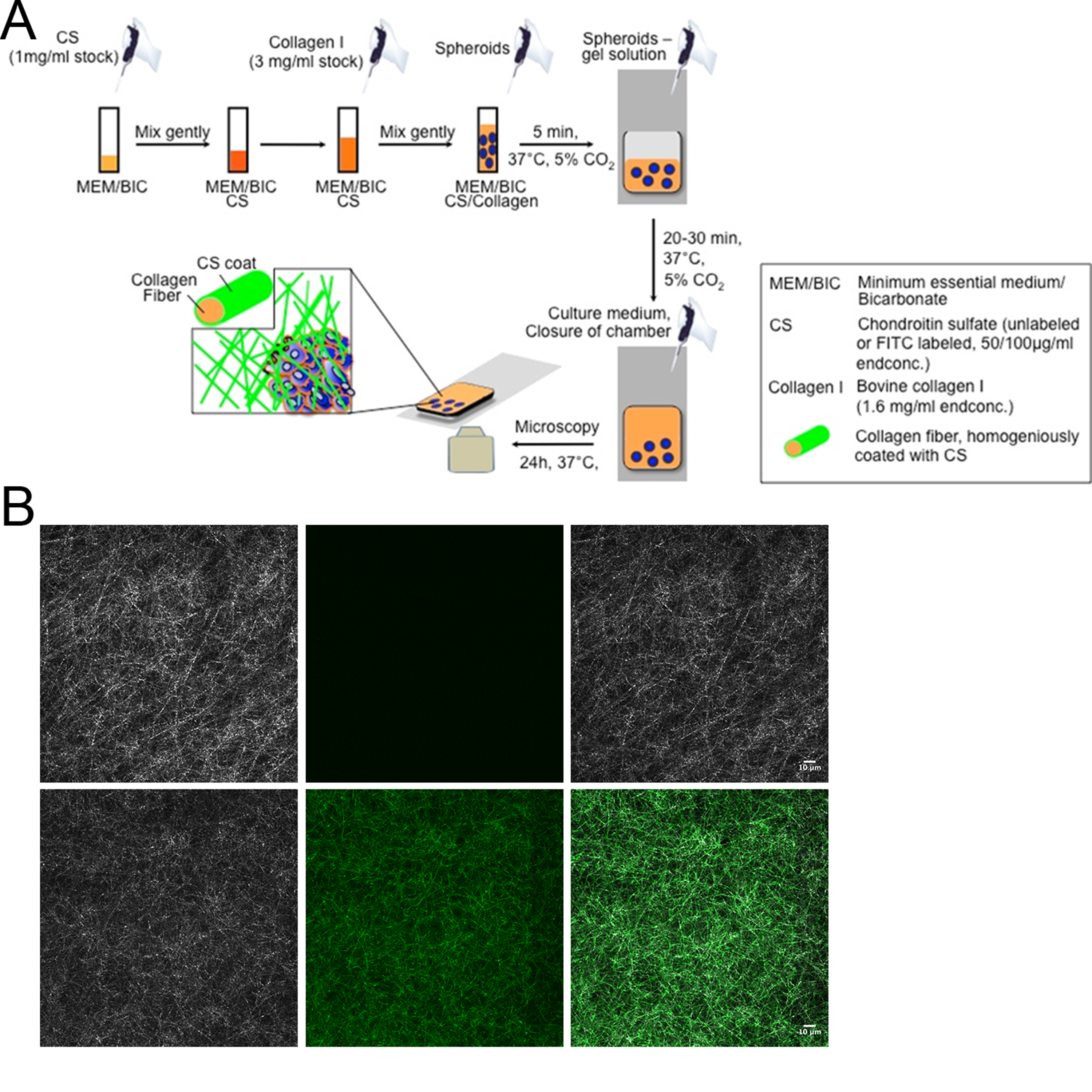

Supplement: Information S2 — Generation of 3D collagen matrices with homogeneously coated chondroitin sulfate E chains, and culturing of SKOV3 spheroids for cell migration studies. A) The spheroids were embedded into a matrix of type I collagen as described previously [54], but with an additional step for administration of the chondroitin sulfate E (CSE, 1 mg/ml) into the minimum essential medium (MEM)/bicarbonate (BIC) solution before adding the bovine type I collagen. SKOV3 spheroids in DMEM medium were transfered into the collagen I/chondroitin sulfate E solution reaching a final concentration of collagen-spheroid suspension of 1.67 mg/mI. The suspension was quickly pre-polymerized for 5 minutes at 37°C, 5% CO2 and eventually allowed to polymerize at 37°C for 20–30 min (5% C02) in a self-constructed cell migration chamber [54]. The type I collagen–chondroitin sulfate matrices were analyzed by using an Olympus FV1000 confocal laser scanning microscope excitation at 488 nm and emission detection of 520/50 nm (for FITC-labeled chondroitin sulfate) and confocal reflection contrast was used for detection of collagen fibers. For that, laser light (633 nm) at a low intensity was introduced into the sample. B) Confocal microscopy showing matrix decoration with chondroitin sulfate E (CSE). Upper row; non-decorated type I bovine collagen matrix. Left: Collagen reflection (white), middle: Background (green (FITC) channel), right: Overlay of reflection and background signal. Lower row; CSE-decorated bovine collagen I matrix. Left: Collagen reflection, middle: CSE-FITC (green (FITC) channel), right: Overlay of reflection and CSE signal. (TIF) [file pone.0111806.s002.tif]
